# Supplementary material for: The phosphorylated pathway of serine biosynthesis affects sperm, embryo, and sporophyte development, and metabolism in Marchantia polymorpha
Source: Commun Biol. 2024 Jan 24;7:102. doi: 10.1038/s42003-023-05746-6 (PMC10808223; doi:10.1038/s42003-023-05746-6)
Supplement: Supplementary file 2 — Supplementary Information [file 42003_2023_5746_MOESM2_ESM.pdf]

The phosphorylated pathway of serine biosynthesis affects sperm, embryo, and sporophyte development, and metabolism in *Marchantia polymorpha*

Mengyao Wang[1,2], Hiromitsu Tabeta[1,3,4, #], Kinuka Ohtaka[1,2,5 #], Ayuko Kuwahara[1], Ryuichi Nishihama[6,7], Toshiki Ishikawa[8], Kiminori Toyooka[1], Mayuko Sato[1], Mayumi Wakazaki[1], Hiromichi Akashi[1], Hiroshi Tsugawa[1,9], Tsubasa Shoji[1], Yozo Okazaki[1,10], Keisuke Yoshida[11], Ryoichi Sato[1], Ali Ferjani [4], Takayuki Kohchi[6], Masami Yokota Hirai[1, 2*]

[1.] RIKEN Center for Sustainable Resource Science, Yokohama, Japan

[2.] Graduate School of Bioagricultural Sciences, Nagoya University, Nagoya, Japan

[3.] Graduate School of Arts and Sciences, The University of Tokyo, Tokyo, Japan

[4.] Department of Biology, Tokyo Gakugei University, Tokyo, Japan

[6.] Graduate School of Biostudies, Kyoto University, Kyoto, Japan

[8.] Graduate School of Science and Engineering, Saitama University, Saitama, Japan

[9.] Department of Biotechnology and Life Science, Tokyo University of Agriculture and Technology, Tokyo, Japan

[10.] Graduate School of Bioresource, Mie University, Tsushi, Japan

[11.] Institute of Innovative Research, Tokyo Institute of Technology, Tokyo, Japan

Present address

[5.] Department of Chemical and Biological Sciences, Japan Women's University, Tokyo, Japan

[7.] Department of Applied Biological Science, Tokyo University of Science, Tokyo, Japan

#These authors contributed to the work equally and should be regarded as co-second authors.

***Corresponding author:**

Masami Yokota Hirai

RIKEN Center for Sustainable Resource Science

Phone: +81-045-503-7040, Fax: +81-045-503-9489

E-mail: masami.hirai@riken.jp

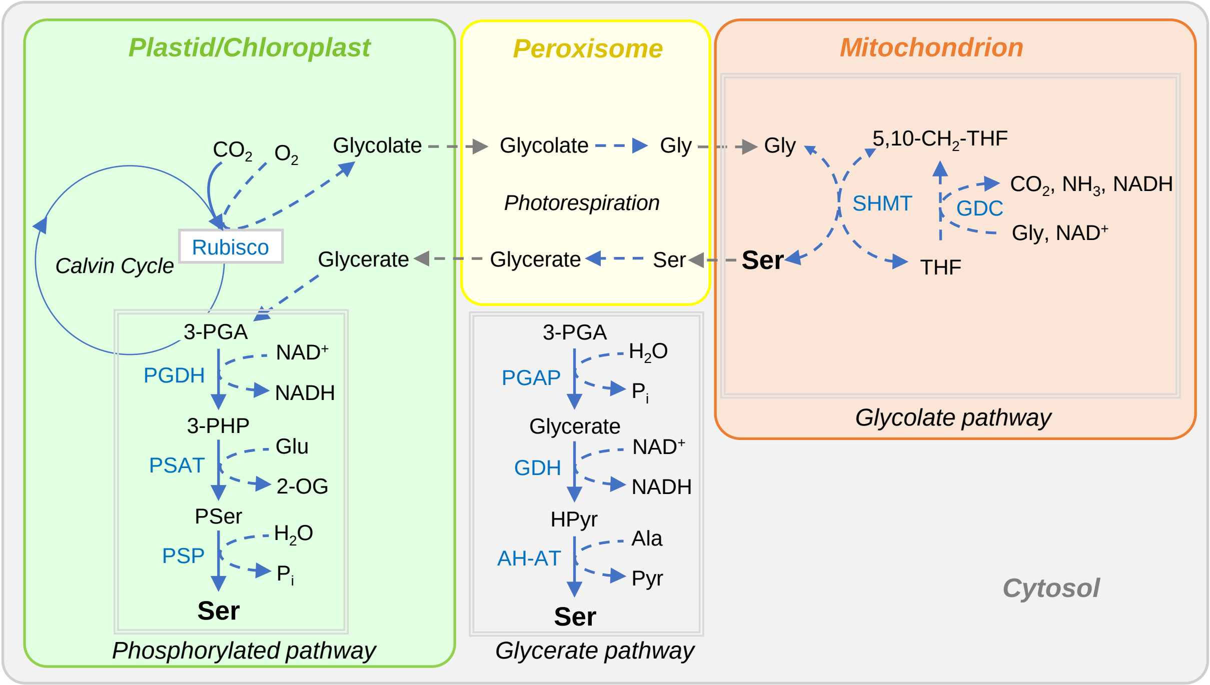

Supplementary Figure 1. Serine biosynthesis pathways in plants.

In plants, serine can be synthesized via three pathways: phosphorylated, glycerate, and glycolate pathways. PGDH, 3-phosphoglycerate dehydrogenase; PSAT, 3-phosphoserine aminotransferase; PSP, 3-phosphoserine phosphatase; PGAP, 3-phosphoglycerate phosphatase; GDH, glycerate dehydrogenase; AH-AT, alanine-hydroxypyruvate aminotransferase; GGAT, glyoxylate glutamate aminotransferase; GDC, glycine decarboxylase complex; SHMT, serine hydroxymethyl transferase; 3-PGA, 3-phosphoglycerate; 3-PHP, 3-phosphohydroxypyruvate; Pser, 3-phosphoserine; Ser, serine; Hpyr, hydroxypyruvate; Ala, alanine; Pyr, pyruvate; Glu, glutamate; Gly, glycine; 2-OG, 2-oxoglutarate; THF, tetrahydrofolate; 5,10-CH_2-THF, 5,10-methylenetetrahydrofolate.

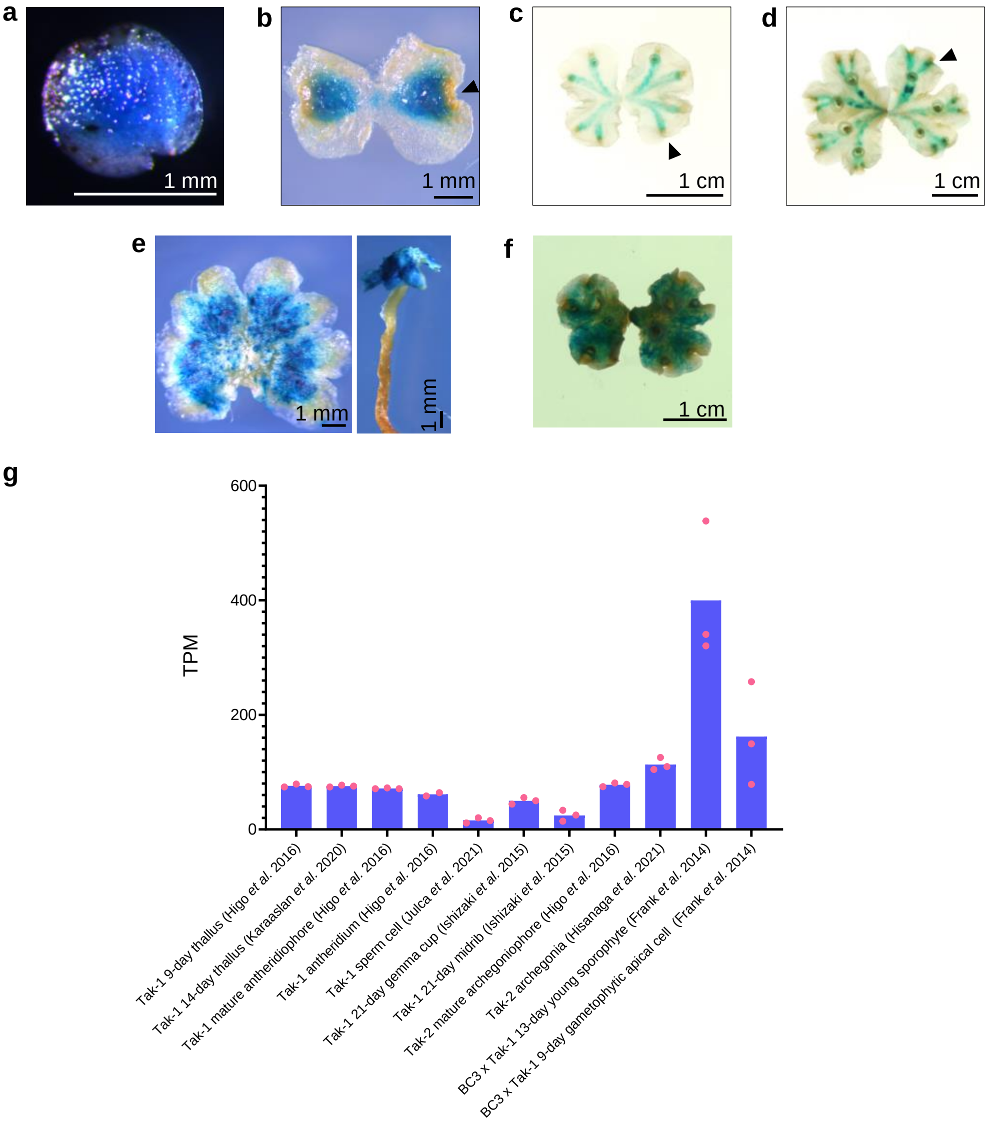

Supplementary Figure 2. Mp*PGDH* expression pattern.

(a)-(e) GUS staining in the gemma (a), 1-week-old thallus (b), 2-week-old thallus (c), 4-week-old thallus (d), and antheridiophore (e) of transgenic plants expressing the GUS reporter gene under the control of Mp*PGDH* promoter grown under L/D conditions in ambient CO_2 (sampled during light exposure). Black arrowheads in (b), (c), and (d) indicate apical notches. (f) GUS staining in 2-week-old thallus grown under L/D condition in high CO_2. **(g)** *In silico* expression analysis using the Expression Database for *M. polymorpha*.

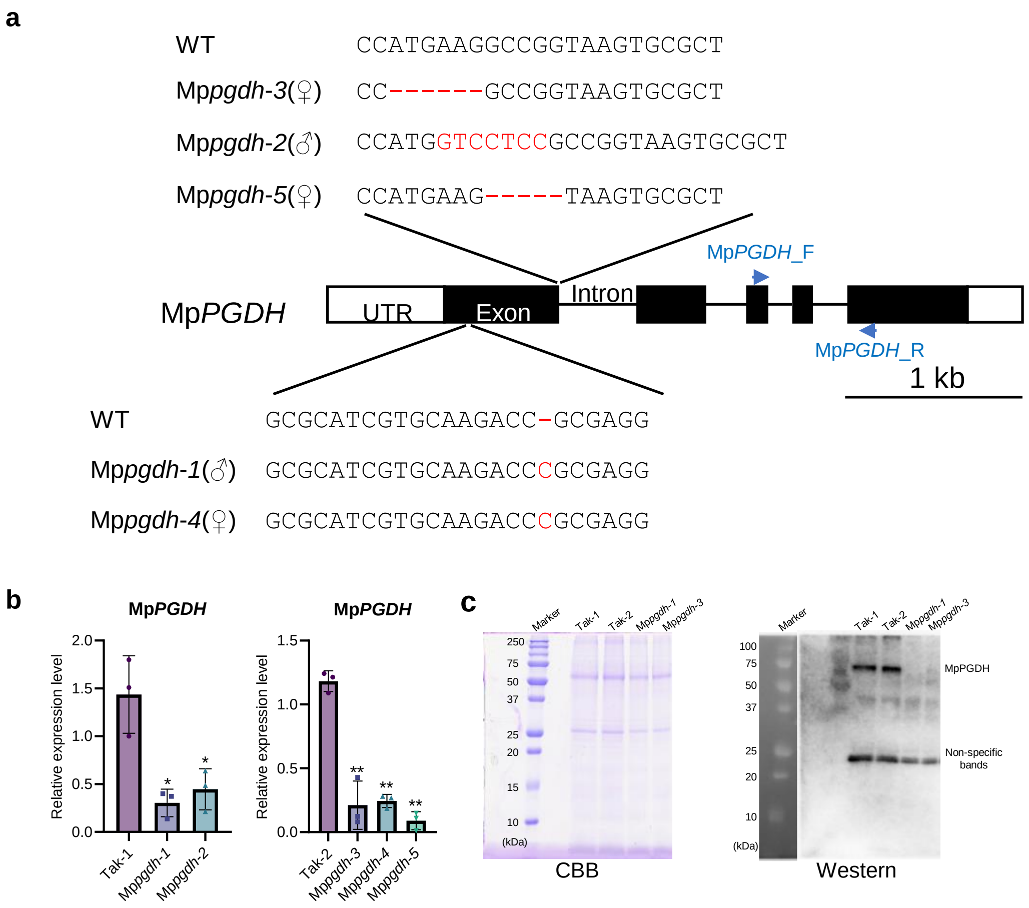

Supplementary Figure 3. Construction of Mp*PGDH* knockout lines.

(a) Mp*PGDH* is a 3902-bp single gene. Mp*PGDH* knockout mutants were constructed using CRISPR/Cas9 system. Two male and three female mutants were selected for the analysis. Mp*pgdh-1* and Mp*pgdh-4* had a 1-bp insertion in the first exon. Mp*pgdh-2* has a 7-bp insertion, whereas Mp*pgdh-3* and Mp*pgdh-5* had 6-bp and 5-bp deletion, respectively, at the border between the first exon and the first intron. The blue arrows show the primers used to check Mp*PGDH* transcript levels in the mutants. **(b)** Mp*PGDH* transcript level in selected mutants. Tak-1 and Tak-2 are male and female wild-type lines, respectively. Mp*ACT1* was used as an internal control. Data are presented as the means ± SD of three biological replicates (n = 3). Asterisks indicate statistically significant differences (Student's *t*-test, $*p < 0.05$, $**p < 0.01$). **(c)** Western blot analysis of MpPGDH protein in wild types and mutants. Coomassie brilliant blue (CBB) staining revealed the total loading quantity of the protein samples. Uncropped images are attached as Supplementary Figure 18.

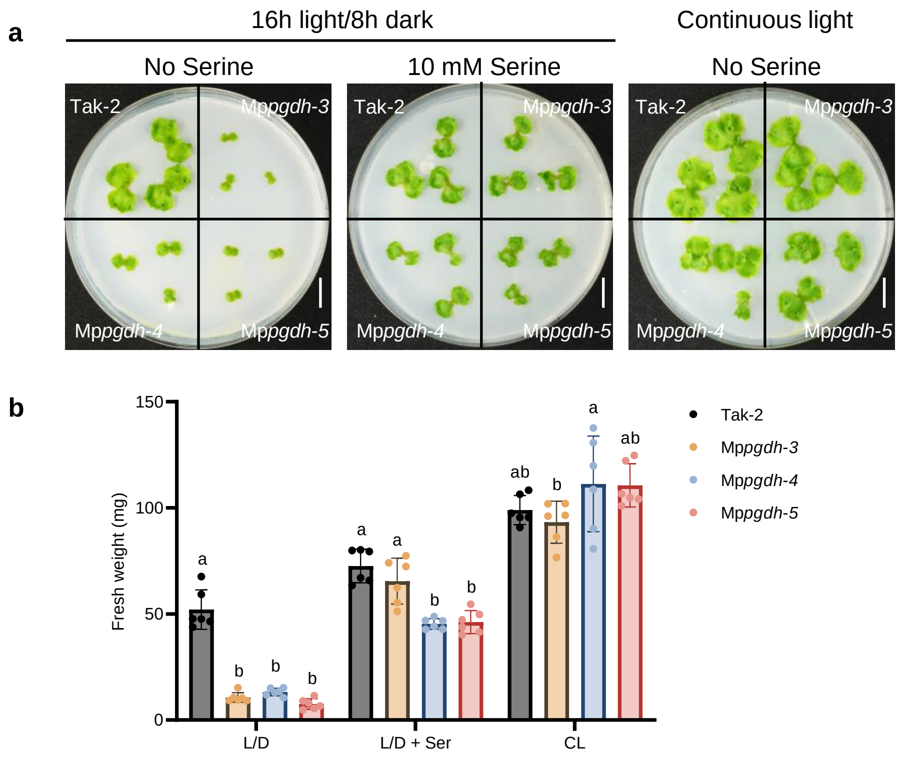

Supplementary Figure 4. Thallus growth in the female Mp*pgdh* mutants.

(a) Plants grown on ½ B5 agar medium for 14 days with or without serine supplementation under 16h light/8h dark (L/D) or continuous light (CL) conditions. Scale bars = 1 cm. **(b)** The fresh weights of Mp*pgdh-3*, Mp*pgdh-4*, Mp*pgdh-5*, and wild-type Tak-2. Data represent means s ± SD of six biological replicates (n = 6). One-way ANOVA followed by Tukey's test ($p < 0.01$) was performed in each group; columns with the same letter are not significantly different.

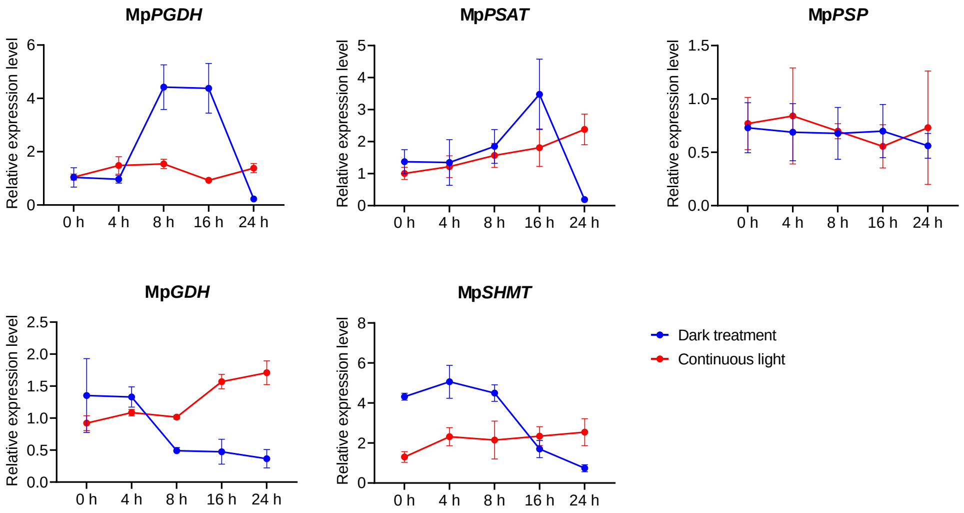

Supplementary Figure 5. Expression of serine biosynthesis pathway genes in the dark.

The 14-day-old Tak-1 thalli grown under the CL condition were transferred to the dark condition or continued to grow under light condition. Total RNA was extracted from Tak-1 at 0, 4, 8, 16, and 24 h after transfer. Two thalli were used as one sample. The expressions of Mp*PGDH*, Mp*PSAT*, Mp*PSP*, Mp*GDH*, and Mp*SHMT* were determined by qRT-PCR. The gene expression was normalized against that of Mp*ACT1*. Data represent means ± SD of three biological replicates (n = 3).

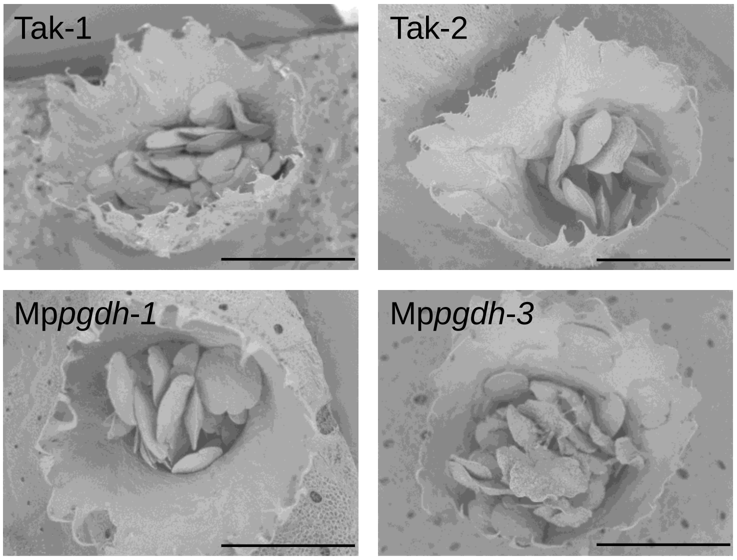

Supplementary Figure 6. Surface scanning electron microscope images of gemma cups.

Plants were grown under L/D conditions for 14 days after germination. Top views of the gemma cups with gemmae inside were taken using a scanning electron microscope. Tak-1 and Tak-2 are the male and female wild type, respectively. Scale bars = 1 mm.

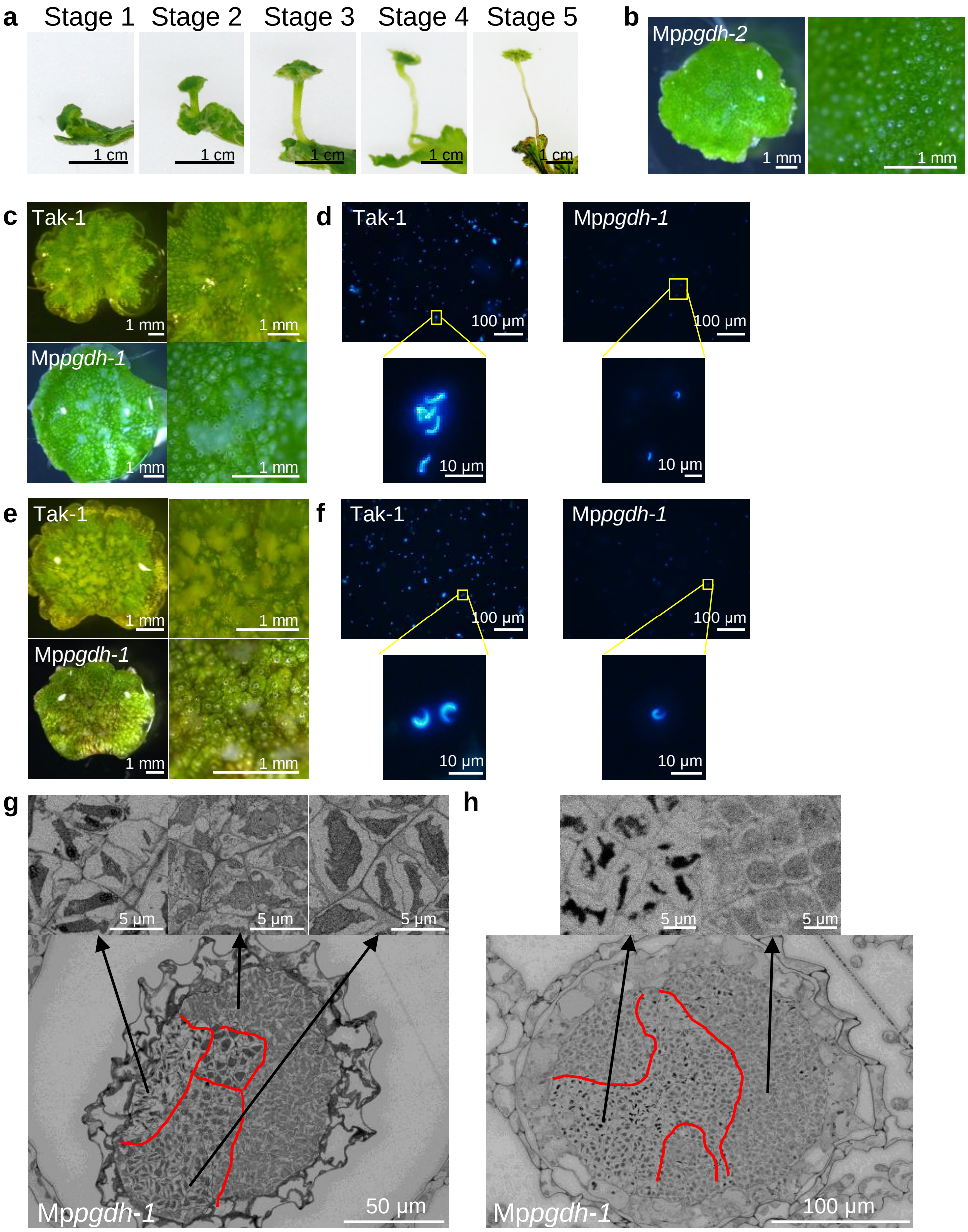

Supplementary Figure 7. Abnormal sperm development in the male Mp*pgdh* mutants.

(a) Five stages of antheridiophore development in Tak-1. **(b)** Sterile phenotype of Mp*pgdh-2*. No sperm masses were discharged from the antheridial pores of Mp*pgdh-2* grown under L/D conditions, after dropping water on the dorsal surface of the antheridiophores. **(c)–(f)** Sperm development under 16-h light/8-h dark (L/D) + serine (c, d) and continuous light (CL) (e, f) conditions. (c, e) Discharge of sperm masses after dropping 50 µL water on the dorsal surface of antheridial receptacles. (d, f) Fluorescent staining of the cells. The cells in 10 µL water from (c) and (e) were visualized via Hoechst staining. **(g), (h)** FE-SEM images of antheridium and spermatogenous cells at different stages of cell division in Mp*pgdh-1* under L/D + serine (g) and CL (h) conditions. The bottom panels show one antheridium. Red lines indicate the edges of different cell areas. The upper panels indicate enlarged images showing the shape of the cells in each area.

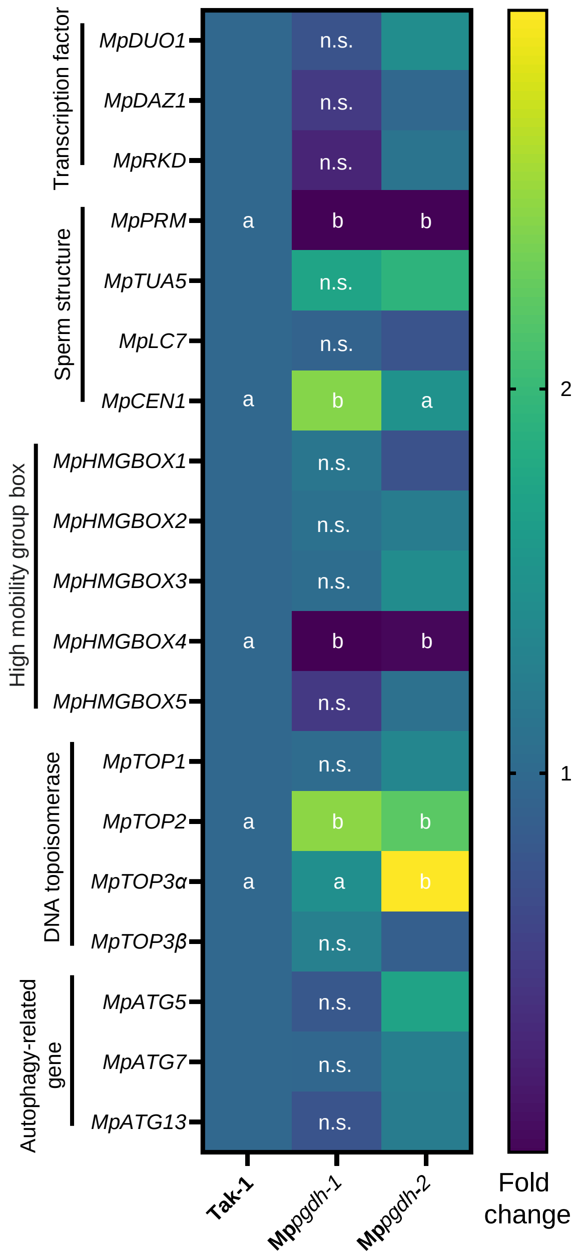

Supplementary Figure 8. Expression of the spermatogenesis-related genes in antheridial receptacles.

The transcript levels of the genes with known functions in spermatogenesis were detected in the antheridial receptacles of Tak-1, Mp*pgdh-1*, and Mp*pgdh-2* grown under L/D conditions using real-time polymerase chain reaction (qRT-PCR) (n = 3). Mp*ACT1* was used as an internal control. Fold change to the expression level in Tak-1 was calculated and presented as a heatmap. Different letters indicate significant differences between the lines. Columns with the same letter are not significantly different (Tukey's test following ANOVA, $p < 0.05$, n.s., no significant difference). DUO1, DUO POLLEN 1; DAZ1, DUO1-ACTIVATED ZINC FINGER1; RKD, RWP-RK domain containing transcription factor; PRM, PROTAMINE-LIKE protein; TUA5, ALPHA-TUBULIN 5; LC7, DYNEIN LIGHT CHAIN 7; CEN1, CENTRIN 1; HMGBOX, High mobility group box protein; TOP, DNA topoisomerase; ATG, Autophagy-related gene.

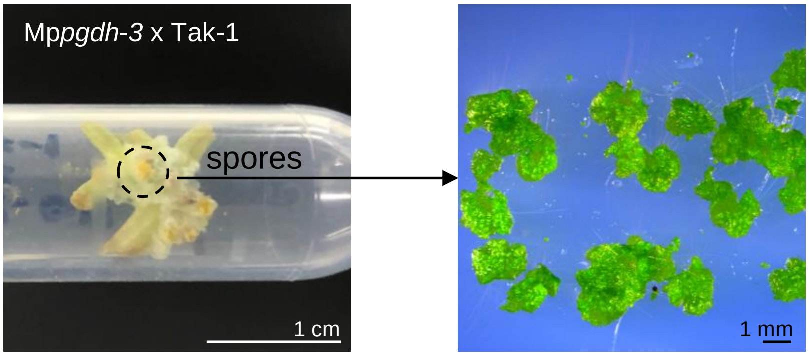

Supplementary Figure 9. Formation of viable spores in the Mp*pgdh-3* x Tak-1 cross under continuous light conditions.

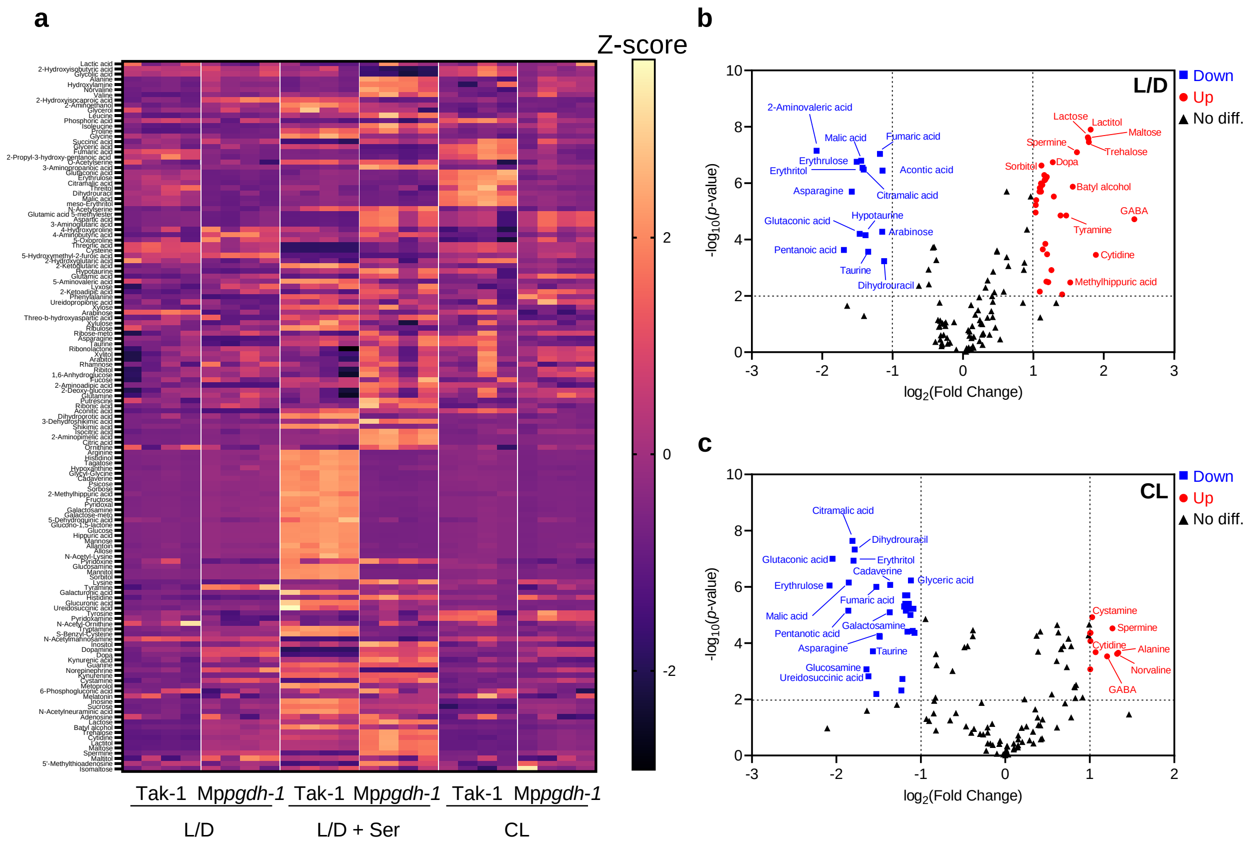

Supplementary Figure 10. Metabolome and differentially accumulated metabolites in 14-day-old thalli of Mp*pgdh-1*.

(a) Heatmap of all quality-filtered metabolites in Tak-1 and Mp*pgdh-1* grown under 16 h light/8 h dark (L/D), L/D + serine, and continuous light (CL) conditions. The color bar on the right side of the heatmap indicates the Z-scores of the average content relative to those of the quality control samples (n = 4). **(b), (c)** Volcano plot showing the differentially accumulated metabolites (DAMs) in Mp*pgdh-1* under L/D (b) and CL (c) conditions. Red dots and blue squares represent significantly increased (*p*-value < 0.01, fold change > 2) and decreased (*p*-value < 0.01, fold change < 0.5) metabolites, respectively, in Mp*pgdh-1*. Black triangles represent no significant differences between Tak-1 and Mp*pgdh-1*.

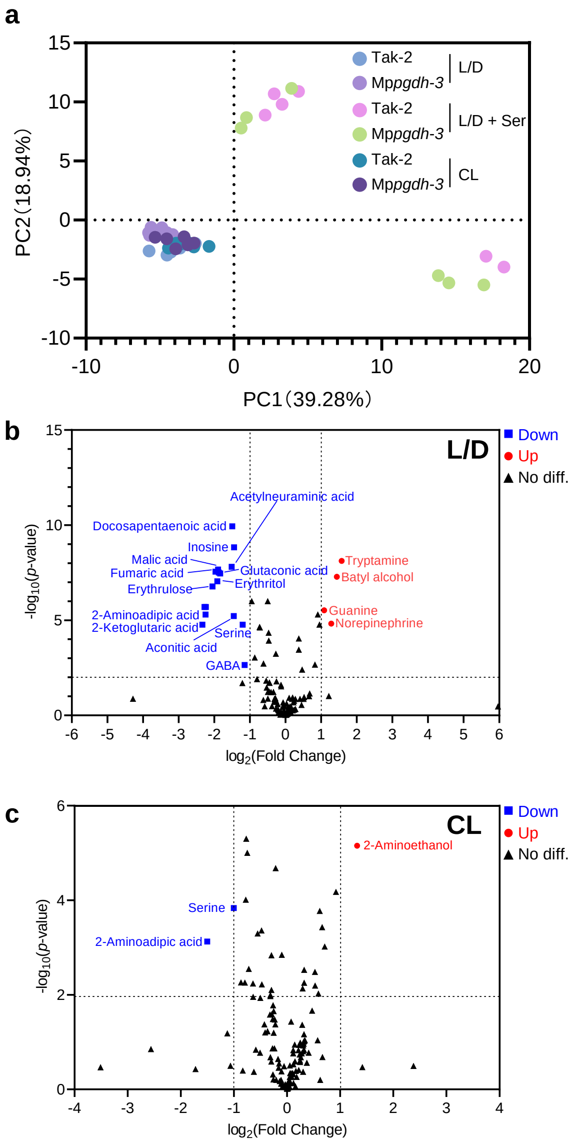

Supplementary Figure 11. Changes in metabolome in 14-day-old thalli of Mp*pgdh-3*.

(a) PCA score plot of Tak-2 and Mp*pgdh*-3 thallus samples grown under L/D, L/D + serine, and CL conditions (n = 6). **(b), (c)** Volcano plot showing the DAMs in thalli of Mp*pgdh-3* under L/D (b) and CL (c) conditions. Red dots and blue squares represent significantly increased (*p*-value < 0.01, fold change > 2) and decreased (*p*-value < 0.01, fold change < 0.5) metabolites, respectively, in Mp*pgdh-3*. Black triangles represent no significant differences between Tak-2 and Mp*pgdh-3*.

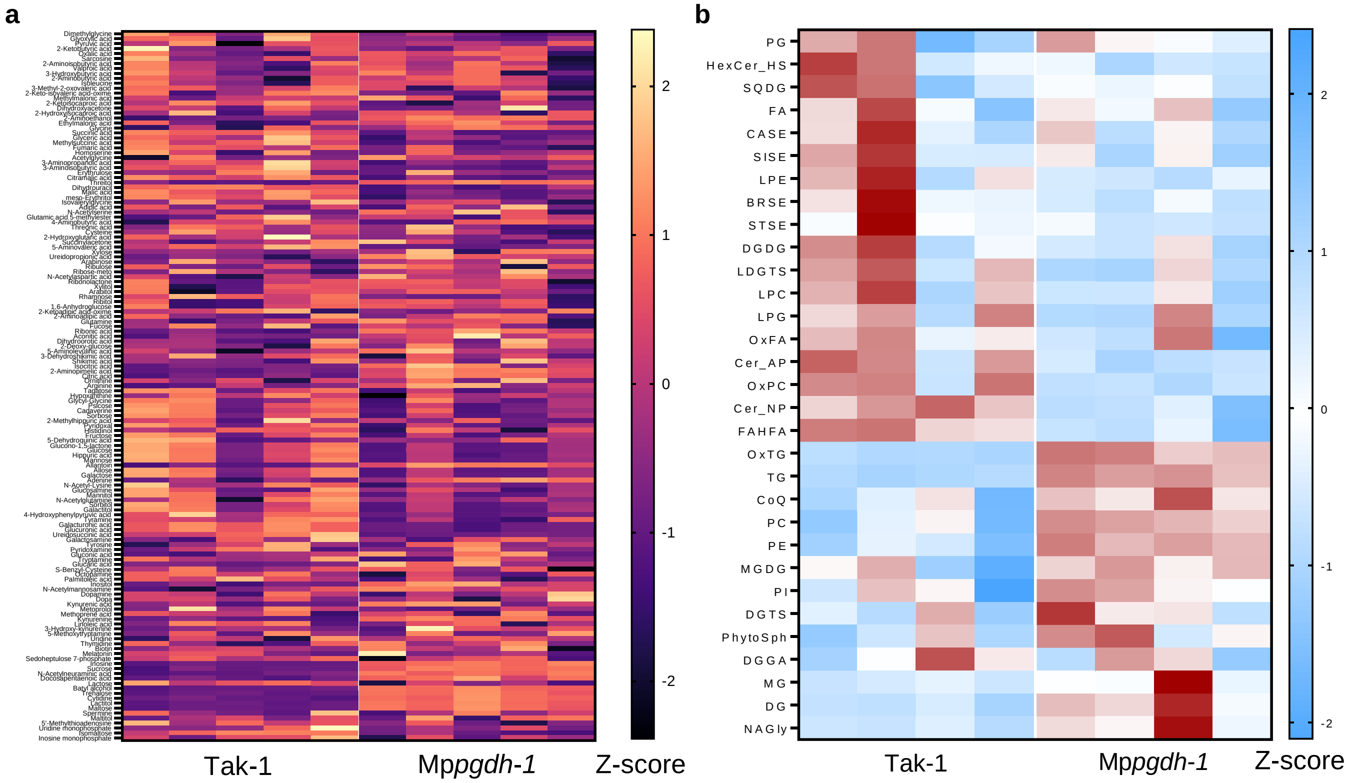

Supplementary Figure 12. Metabolome and lipidome in antheridial receptacle.

(a), (b) Heatmap of all quality-filtered metabolites (a) and lipid classes (b) in antheridial receptacles of Tak-1 and Mp*pgdh*-1 grown under 16-h light/8-h dark (L/D) conditions. The color bar on the right side of the heatmap indicates the Z-scores of the average content relative to those of the quality control samples.

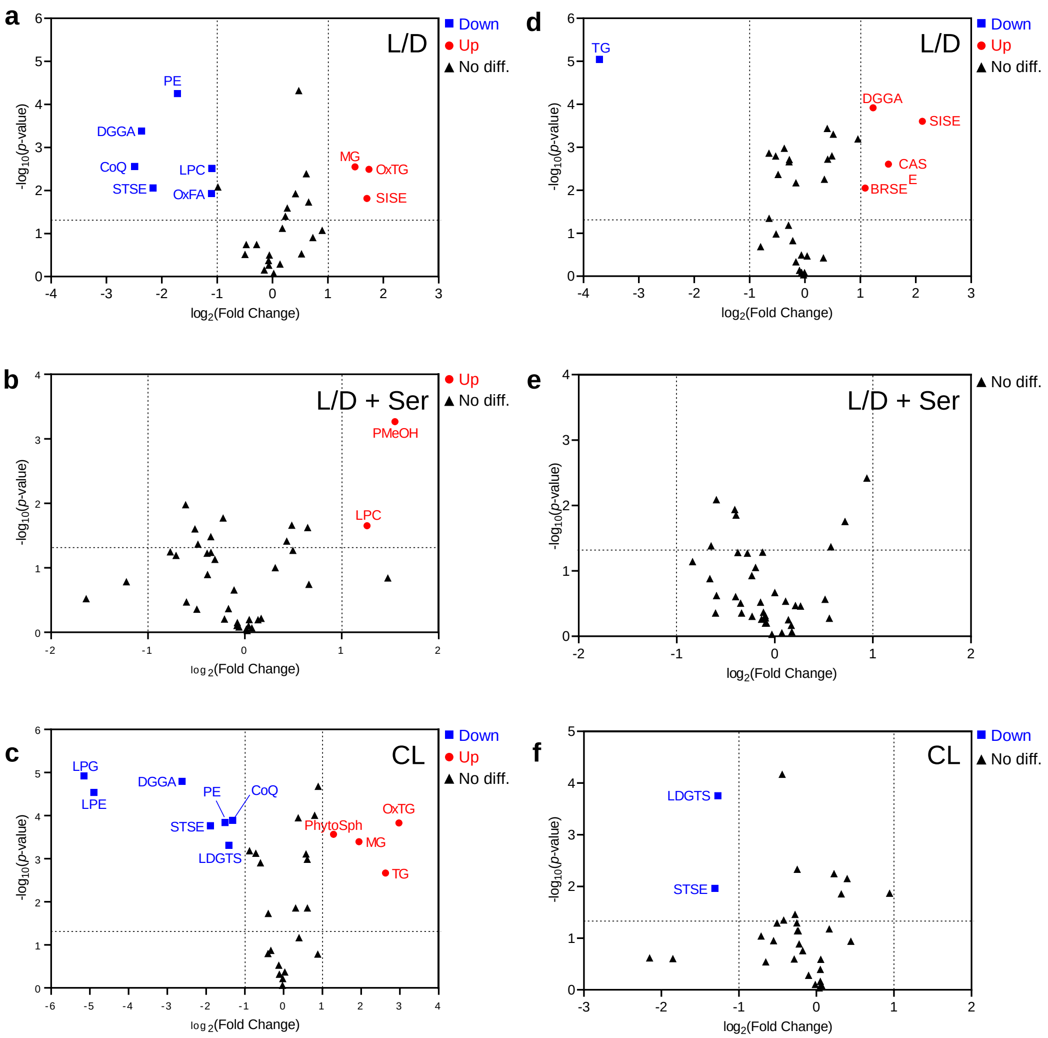

Supplementary Figure 13. Differentially accumulated lipid classes in 14-day-old thalli of Mp*pgdh* mutants. (a)-(c) Volcano plot showing the differentially accumulated lipid classes in thalli of Mp*pgdh-1* under L/D (a), L/D + serine (b), and CL (c) conditions. **(d)-(f)** Volcano plot showing the differentially accumulated lipid classes in thalli of Mp*pgdh-3* under L/D (d), L/D + serine (e), and CL (f) conditions. Red dots and blue squares represent significantly increased (*p*-value < 0.05, fold change > 2) and decreased (*p*-value < 0.05, fold change < 0.5) lipid classes, respectively (n = 3). Black triangles represent no significant differences between mutants and wild types.

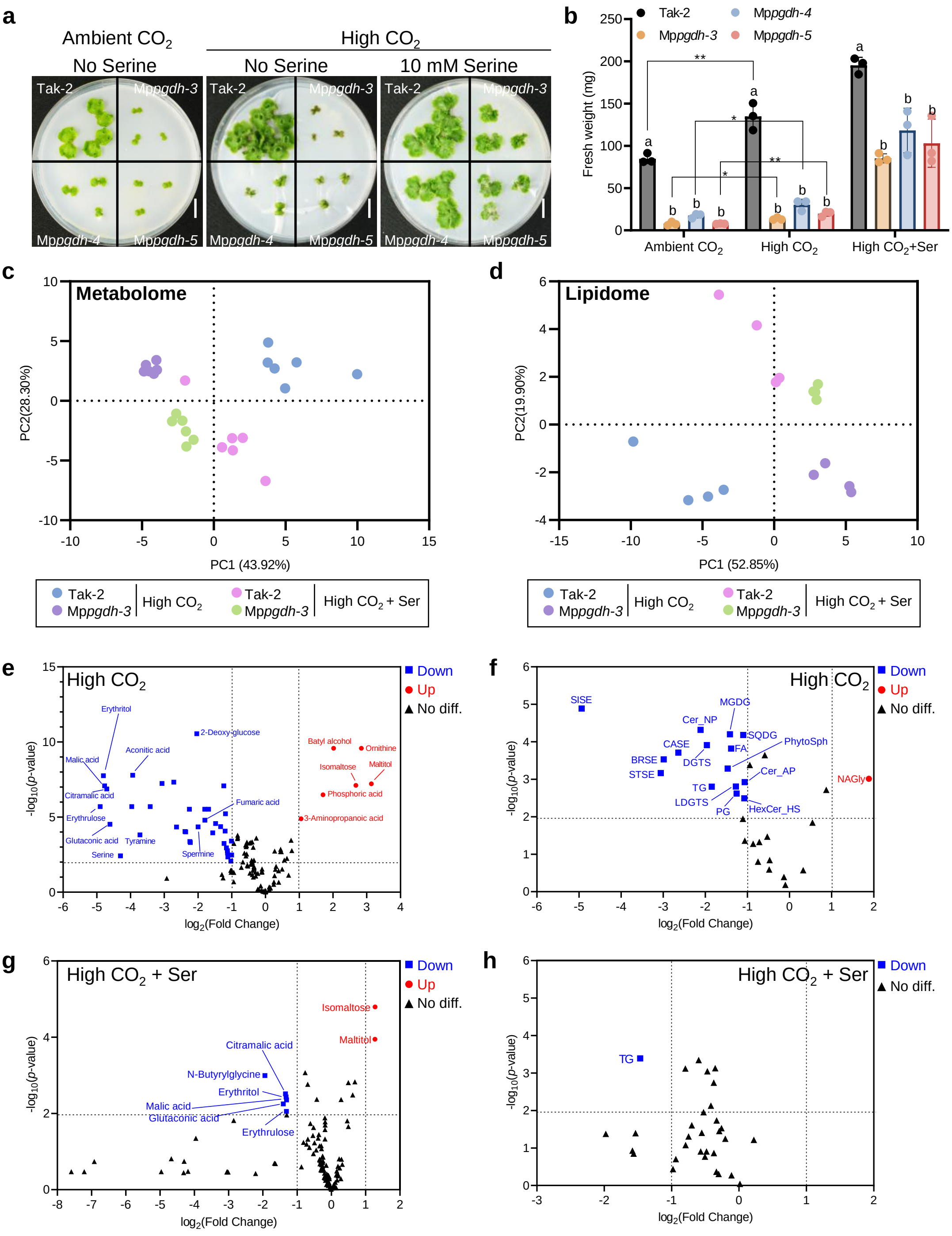
a
Ambient CO2
High CO2
No Serine
No Serine
10 mM Serine
Tak-2
Mppgdh-3
Mppgdh-4
Mppgdh-5
b
Tak-2
Mppgdh-3
Mppgdh-4
Mppgdh-5
Fresh weight (mg)
Ambient CO2
High CO2
High CO2+Ser
c
Metabolome
PC2(28.30%)
PC1 (43.92%)
d
Lipidome
PC2(19.90%)
PC1 (52.85%)
Tak-2
Mppgdh-3
High CO2
High CO2 + Ser
e
High CO2
Down
Up
No diff.
-log10(p-value)
log2(Fold Change)
Erythritol
2-Deoxy-glucose
Aconitic acid
Malic acid
Citramalic acid
Erythrulose
Glutaconic acid
Tyramine
Serine
Spermine
Fumaric acid
Batyl alcohol
Ornithine
Isomaltose
Maltitol
Phosphoric acid
3-Aminopropanoic acid
f
High CO2
SISE
MGDG
Cer_NP
CASE
SQDG
FA
PhytoSph
BRSE
DGTS
STSE
Cer_AP
TG
LDGTS
PG
HexCer_HS
NAGly
g
High CO2 + Ser
Isomaltose
Maltitol
Citramalic acid
N-Butyrylglycine
Erythritol
Malic acid
Glutaconic acid
Erythrulose
h
High CO2 + Ser
TG

Supplementary Figure 14. Growth and metabolic phenotypes of female Mp*pgdh* mutants under high CO_2 conditions.

(a) Plants grown on ½ B5 agar medium for 14 days under L/D conditions in ambient CO_2 (400 ppm) or high CO_2 (3000 ppm) with or without serine supplementation. Scale bars = 1 cm. **(b)** The fresh weight of Tak-2 and Mp*pgdh* mutants. Data are presented as means ± SD of three biological replicates (n = 3). One-way ANOVA followed by Tukey's test ($p < 0.05$) was performed in each growth condition; columns with the same letter indicate no significant differences. Student's *t*-test was performed in each line grown under ambient CO_2 and high CO_2 conditions. Asterisks indicate statistically significant differences (Student's *t*-test, *p <0.05, **p < 0.01). **(c), (d)** PCA score plots of metabolome (c) (n = 6) and lipidome (d) (n = 4) data of Tak-2 and Mp*pgdh-3* thalli grown under high CO_2 conditions with or without serine supplementation. **(e)-(h)** Volcano plot showing DAMs (e, g) and DALCs (f, h) in Mp*pgdh-3* under the two growth conditions. Red dots and blue squares represent significantly increased (*p*-value < 0.01, fold change > 2) and decreased (*p*-value < 0.01, fold change < 0.5) metabolites/lipid classes, respectively, in Mp*pgdh-3*. Black triangles represent no significant differences between Tak-2 and Mp*pgdh-3*.

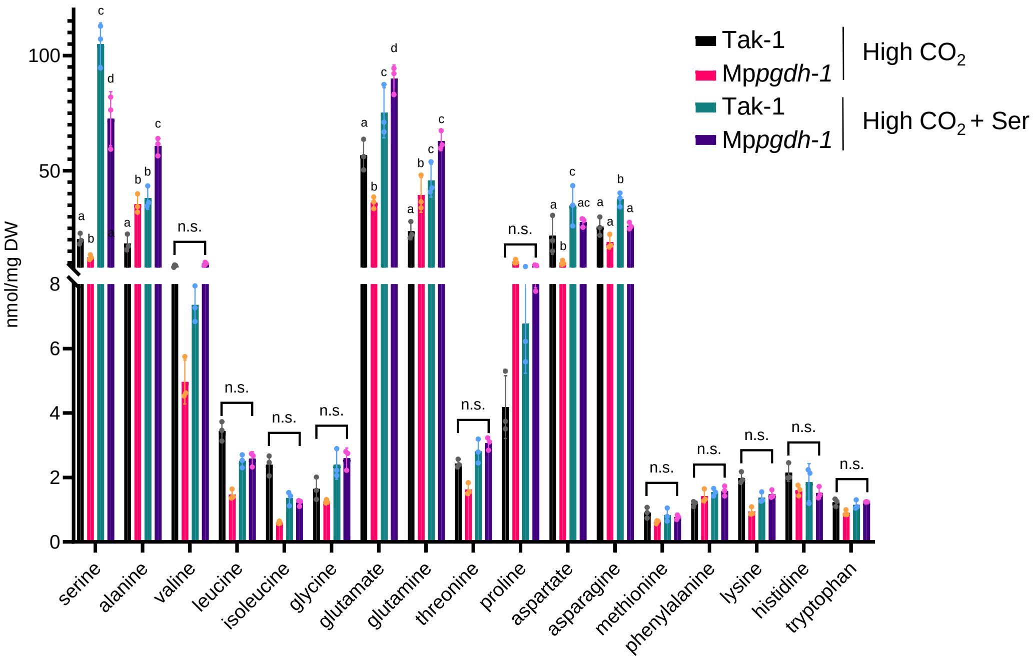

Supplementary Figure 15. Free amino acid contents in thalli under high CO_2 conditions.

The thalli were grown under L/D conditions in high CO_2 with or without serine supplementation and the free amino acid contents were measured using gas chromatography–quadrupole mass spectrometry. Data represent means ± SD of three biological replicates (n = 3). Means with different letters are significantly different (Tukey's test following ANOVA, $p < 0.05$; n.s., no significant difference).

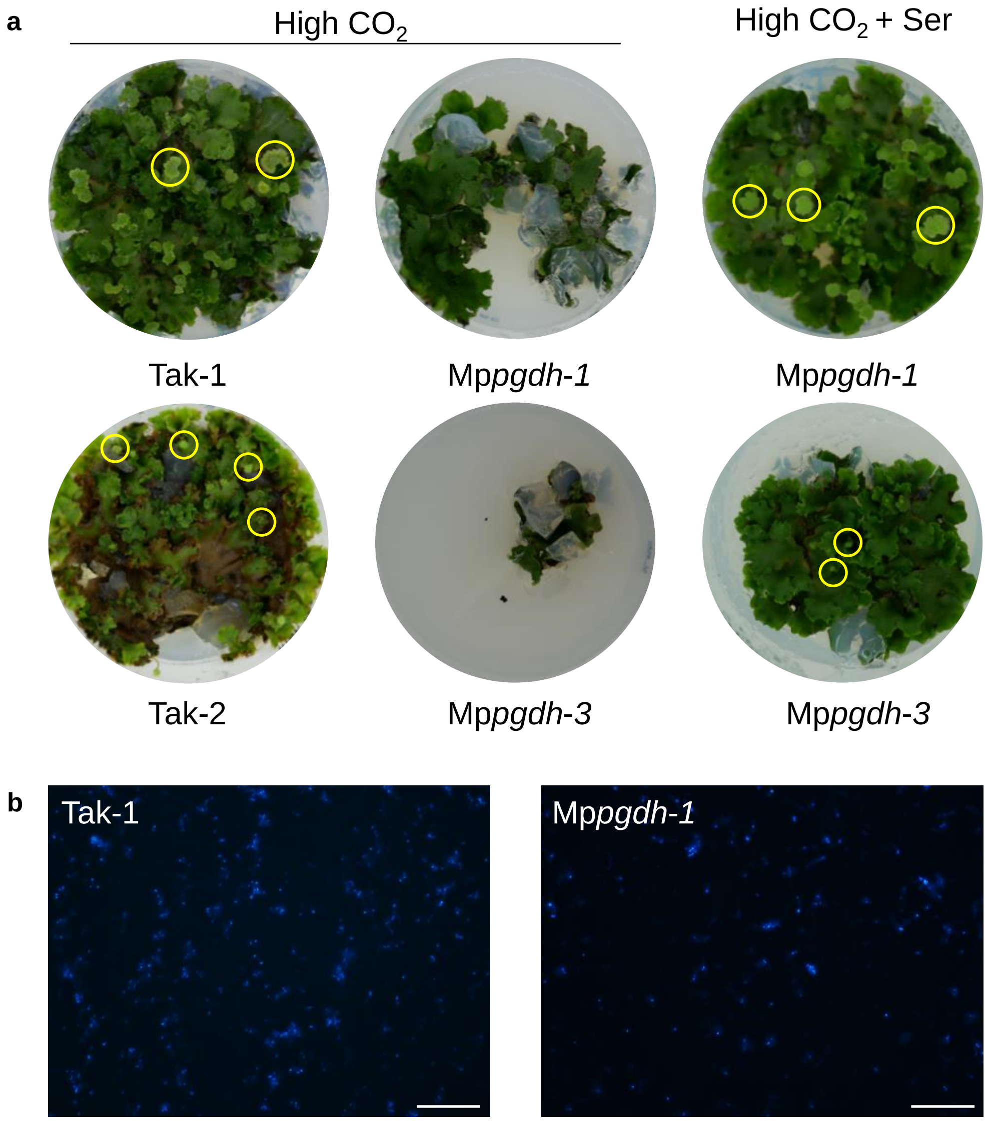

Supplementary Figure 16. Sexual reproduction of the mutants under high CO_2 conditions.

(a) The development of sexual reproductive branches of plants grown in high CO_2 under L/D conditions for 2 months. The yellow circle indicates sexual reproductive branches. **(b)** Fluorescent staining of the cells from antheridial receptacles of the plants grown under high CO_2 conditions with serine supplement. Scale bars = 100 μm.

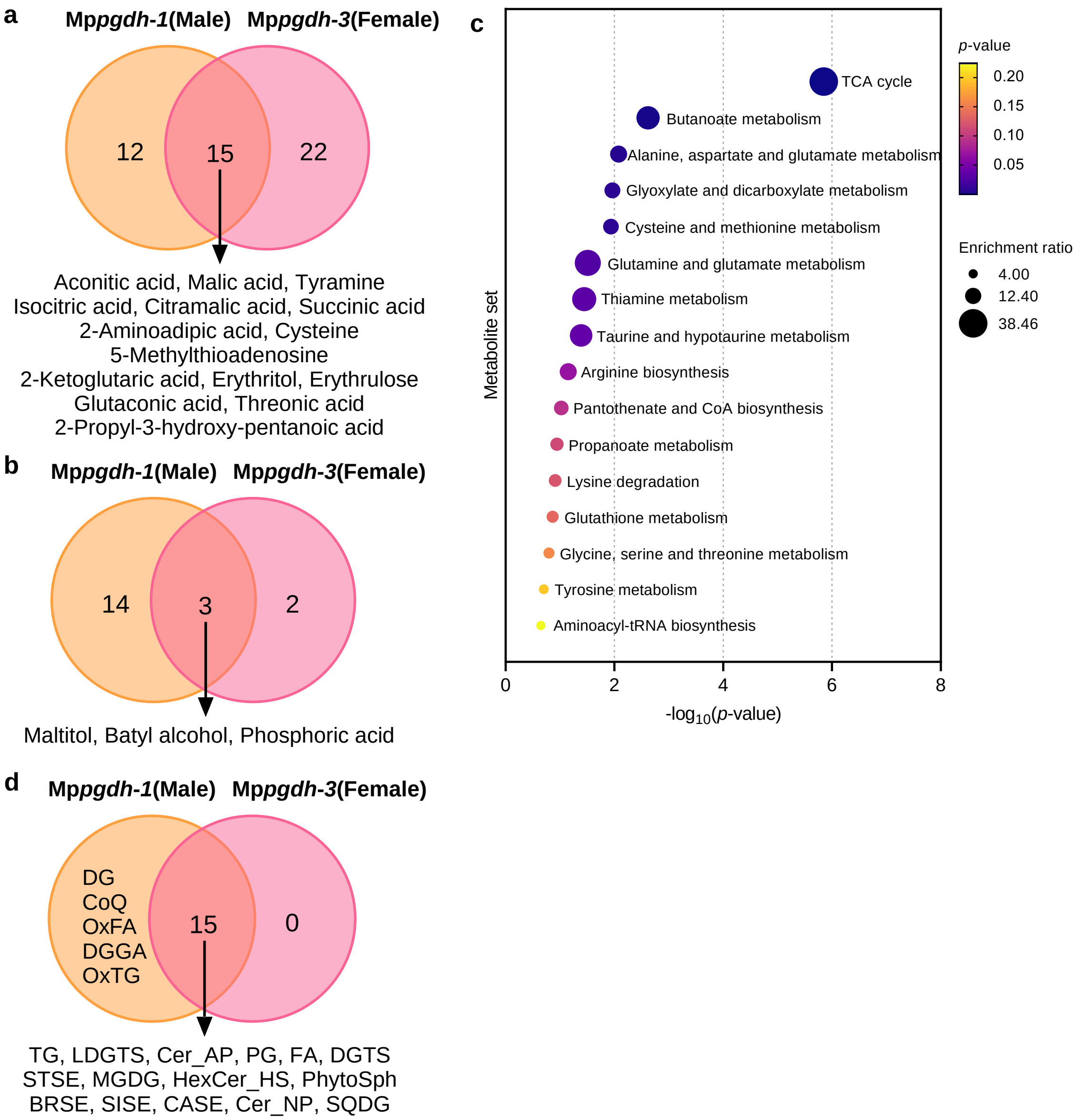

Supplementary Figure 17. Changes in metabolome and lipidome in 14-day-old thalli of the Mp*pgdh* mutants grown under high CO_2 conditions.

(a), (b) Venn diagrams showing the number of significantly decreased metabolites (a) and increased metabolites (b) in thalli of Mp*pgdh-1* and Mp*pgdh-3* under high CO_2 conditions. **(c)** KEGG pathway enrichment analysis of common DAMs shown in (a) and (b). Vertical and horizontal axes indicate the metabolite set and the value of $-\log_{10}$(*p*-value), respectively. The bubble size corresponds to the enrichment ratio. The color bar indicates the corrected *p*-value; yellow and navy blue represent higher and lower values, respectively. **(d)** Venn diagram showing the number of significantly decreased lipid classes in thalli of Mp*pgdh-1* and Mp*pgdh-3* under high CO_2 conditions. Abbreviations are defined in Supplementary Table 2.

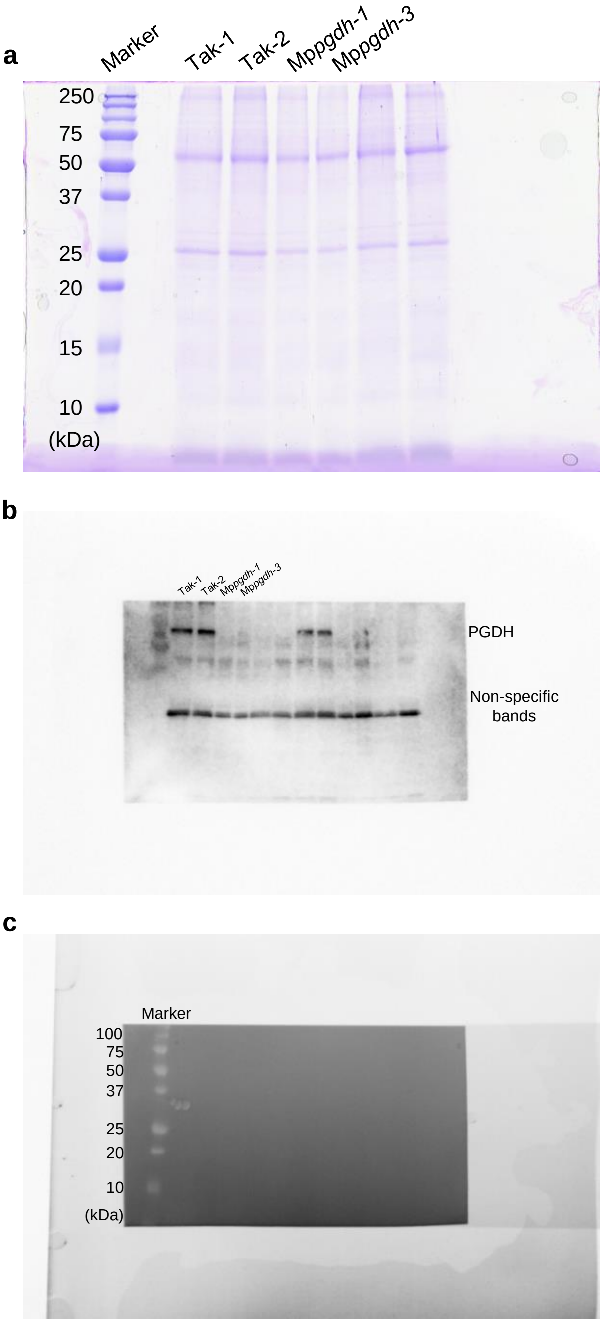

Supplementary Figure 18. Uncropped blots illustrated in supplementary figure 3c.

(a) Uncropped Coomassie Brilliant Blue staining of the SDS-PAGE gel depicted in the left panel of Supplementary Figure 3c. **(b)** Uncropped western blot detected by chemiluminescence as describe in the methods. **(c)** Uncropped blot visualized under visible light to reveal the marker's position. (b) and (c) were captured using the same polyvinylidene difluoride membrane, positioned identically, and with the same amplification ratio.

Supplementary Table 1. Primer pairs used for constructing Mp*PGDH* mutants and *pro*Mp*PGDH*:*GUS* lines.

Purpose	Primer name	Sequence (5'-3')
CRISPR/Cas9 line	Mp*PGDH*_gRNA_f1	CTCGGCGCATCGTGCAAGACCGCG
	Mp*PGDH*_gRNA_r1	AAACCGCGGTCTTGCACGATGCGC
	Mp*PGDH*_gRNA_f2	CTCGAGCGCACTTACCGGCCTTCA
	Mp*PGDH*_gRNA_r2	AAACTGAAGGCCGGTAAGTGCGCT
proMp*PGDH*:*GUS* line	Mp*PGDH*pro_5kb_F	CACAAAATGTCATATAAAAGGAGTAAAAATG
	Mp*PGDH*pro_5kb_R	CATGACCGCGTCTGCCATTTCTCTCCGGGC
	pMpGWB104_r3021	CCCAATCCAGTCCATTAATGCGTGGTCGTG
	pMpGWB104_r4111	AATGTATAATTGCGGGACTCTAATCATAAA
Male line selection	rbm27-F	ACTTTTGCAACAGCGACTTC
	rbm27-R	GCCTGCAATATAGCCTTCAA
Female line selection	rhf73-F	GAACCCGAAACTCAGGTTTT
	rhf73-R	ATAACAGCCAAACGGATCAA

Supplementary Table 2. Primer pairs used for qRT-PCR

Gene	Forward (5'-3')	Reverse (5'-3')
Mp*ACT1*	CAGTGTCTGGATCGGAGGAT	CACCACTCAAACTGGAAGCA
Mp*PGDH*	CGCAGGAAGGAGTTGCAGTAG	TCTCTCGGCCAAAGTGACATAG
Mp*PSAT*	TCTGTCCCCGTGTGTAATGC	GTGCATCGGGTGACAAGAGA
Mp*PSP*	TTGATGCTACCGAACCCACC	ACGGACTGCTACTCCTCCAT
Mp*SHMT*	AAGTACTCCGAGGGTCTCCC	CATTTCGCAGGATCGCAGTG
Mp*GDH*	AATGGCGACATTAGCTGCCT	GAGTCCCAAGACTTTCGCGT
Mp*DUO1*	CTCTTAGTTCGAAGAACAGCGGAG	CACCCACTCTTGAGATCCGGCTTG
Mp*DAZ1*	TGTTTTATCACCCGCCTAGC	AGGCGAACAAAACATTGGAC
Mp*MID*	CACATCATCTGAAGCTGTCAGATCTATC	CTAAACTTCGAACCTTGCGTCCTGG
Mp*RKD*	TCGAGCTTTGGCAATGCATA	TTGCGGATTCCTCGTGACAC
Mp*PRM*	ATCCAGCGGCGTGAGCCAGA	CGAAGAATGCGGAAGACTGA
Mp*TUA5*	GTGCAGATCGACGACAAAGGATC	GAGAATTCTCCGAAGCTCGTGAG
Mp*LC7*	CGTCGACAATGACGGAAATA	TACTGTTGTGCGACCAGGAG
Mp*CEN1*	CCAAAATGGGTGAACGAGAT	ACCATCTCGATCTGCTTCGT
Mp*HMGBOX1*	TACGGGGAAGGCGAAGAAAG	GGCTTGGCGTCTTTGACAAC
Mp*HMGBOX2*	AGAGCAGAAGAAGCTCGCTG	TGACGGGAACCTCAATGGTG
Mp*HMGBOX3*	AGCAGGCTCAGATGACGAAG	GTCATCCGTTGGTGACCCTT
Mp*HMGBOX4*	CGAGCAACAAATCGACGAGG	GCCGAAAGTCATAGTCGGGT
Mp*HMGBOX5*	TGGTCACTGTAAGGGTGGGA	ACAATGCCGTCGTTCCTTCT
Mp*TOP1*	CGAAGAAGAGACCCGCAGTT	AAGAACCCGATTTGGGCGAT
Mp*TOP2*	CACCTACAAAGCGGGTCACT	CTCCGCCTCAAAGTCGGAAT
Mp*TOP3α*	TCAACCGCCACGGTTATTCA	TGCGGAAGTTTCGAGAGCAT
Mp*TOP3β*	CCTTTGACCTCTTCGACGCT	TAATCGCAGGAGCGACCTTC
Mp*ATG5*	CCGGGAGATTACCAATGCGT	GGAACGTCGATCGGTCTTGT
Mp*ATG7*	ATGGAGCCGTCAATGCAAGA	AGTTACGGAGAGGCCATCCT
Mp*ATG13*	TTCTCTTCCGTCTTCGGTGC	GCTGGGGGTAACTGCATCTT

Supplementary Table 3. List of lipid abbreviations

Categories	Abbreviations	Definition
Sterol Lipids	BRSE	Brassicasterol ester
	CASE	Campesterol ester
	SISE	Sitosterol ester
	STSE	Stigmasterol ester
Sphingolipids	PhytoSph	Phytosphingosine
	Cer_AP	Ceramide alpha-hydroxy fatty acid-phytosphingosine
	Cer_NP	Ceramide non-hydroxyfatty acid-phytosphingosine
	HexCer_HS	Hexosylceramide hydroxyfatty acid-sphingosine
Glycerolipids	DG	Diacylglycerol
	DGDG	Digalactosyldiacylglycerol
	MGDG	Monogalactosyldiacylglycerol
	SQDG	Sulfoquinovosyl diacylglycerol
	DGGA	Diacylglyceryl glucuronide
	DGTS	Diacylglyceryl trimethylhomoserine/diacylglyceryl hydroxymethyl-N,N,N-trimethyl-β-alanine
	LDGTS	Lysodiacylglyceryl trimethylhomoserine/Lysodiacylglyceryl hydroxymethyl-N,N,N-trimethyl-β-alanine
	MG	Monoacylglycerol
	TG	Triacylglycerol
	OxTG	Oxidized triglyceride
Glycerophospholipids	PC	Phosphatidylcholine
	OxPC	Oxidized phosphatidylcholine
	LPC	Lysophophatidylcholine
	PG	Phosphatidylglycerol
	LPG	Lysophosphatidylglycerol
	PE	Phosphatidylethanolamine
	LPE	Lysophosphatidylethanolamine
	PI	Phosphatidylinositol
Fatty acyls	FA	Free fatty acid
	OxFA	Oxidized fatty acid
	NAGly	N-acyl glycine
	FAHFA	Fatty acid ester of hydroxyl fatty acid
Prenol Lipids	CoQ	Coenzyme Q
