# Supplementary material for: The phosphorylated pathway of serine biosynthesis affects sperm, embryo, and sporophyte development, and metabolism in Marchantia polymorpha
Source: Commun Biol. 2024 Jan 24;7:102. doi: 10.1038/s42003-023-05746-6 (PMC10808223; doi:10.1038/s42003-023-05746-6)
Supplement: Supplementary file 3 — Description of Additional Supplementary Files [file 42003_2023_5746_MOESM3_ESM.pdf]

Description of Additional Supplementary Files

File name: Supplementary Data 1
Description: Differentially accumulated metabolites and lipid classes in Mppgdh mutants.

File name: Supplementary Data 2
Description: The source data underlying the graphs in the main figures.

File name: Supplementary Data 3
Description: Uncropped Western Blot images in Supplementary Figure 3c.
