# Supplementary material for: The phosphorylated pathway of serine biosynthesis affects sperm, embryo, and sporophyte development, and metabolism in Marchantia polymorpha
Source: Commun Biol. 2024 Jan 24;7:102. doi: 10.1038/s42003-023-05746-6 (PMC10808223; doi:10.1038/s42003-023-05746-6)
Supplement: Supplementary file 5 — Reporting Summary [file 42003_2023_5746_MOESM5_ESM.pdf]

nature portfolio

Corresponding author(s): Masami Yokota Hirai

Last updated by author(s): Dec 6, 2023

Reporting Summary

Nature Portfolio wishes to improve the reproducibility of the work that we publish. This form provides structure for consistency and transparency in reporting. For further information on Nature Portfolio policies, see our Editorial Policies and the Editorial Policy Checklist.

Statistics

For all statistical analyses, confirm that the following items are present in the figure legend, table legend, main text, or Methods section.

n/a	Confirmed	
☐	☒	The exact sample size (*n*) for each experimental group/condition, given as a discrete number and unit of measurement
☐	☒	A statement on whether measurements were taken from distinct samples or whether the same sample was measured repeatedly
☐	☒	The statistical test(s) used AND whether they are one- or two-sided<br>*Only common tests should be described solely by name; describe more complex techniques in the Methods section.*
☒	☐	A description of all covariates tested
☐	☒	A description of any assumptions or corrections, such as tests of normality and adjustment for multiple comparisons
☐	☒	A full description of the statistical parameters including central tendency (e.g. means) or other basic estimates (e.g. regression coefficient) AND variation (e.g. standard deviation) or associated estimates of uncertainty (e.g. confidence intervals)
☐	☒	For null hypothesis testing, the test statistic (e.g. *F*, *t*, *r*) with confidence intervals, effect sizes, degrees of freedom and *P* value noted<br>*Give P values as exact values whenever suitable.*
☒	☐	For Bayesian analysis, information on the choice of priors and Markov chain Monte Carlo settings
☒	☐	For hierarchical and complex designs, identification of the appropriate level for tests and full reporting of outcomes
☒	☐	Estimates of effect sizes (e.g. Cohen's *d*, Pearson's *r*), indicating how they were calculated

Our web collection on statistics for biologists contains articles on many of the points above.

Software and code

Policy information about availability of computer code

Data collection	GCMS solution software (Shimadzu Corporation), MRMPROBS and LOWESS/Spline normalization tools (http://prime.psc.riken.jp/compms/mrmprobs/main.html), MS-DIAL version 4.80 (http://prime.psc.riken.jp/compms/msdial/main.html).
Data analysis	MetaboAnalyst 5.0 (https://www.metaboanalyst.ca/home.xhtml), Webtools (https://bioinformatics.psb.ugent.be/webtools/Venn/), GraphPad Prism 9.0 (https://www.graphpad.com/).

For manuscripts utilizing custom algorithms or software that are central to the research but not yet described in published literature, software must be made available to editors and reviewers. We strongly encourage code deposition in a community repository (e.g. GitHub). See the Nature Portfolio guidelines for submitting code & software for further information.

Data

Policy information about availability of data

All manuscripts must include a data availability statement. This statement should provide the following information, where applicable:

- Accession codes, unique identifiers, or web links for publicly available datasets
- A description of any restrictions on data availability
- For clinical datasets or third party data, please ensure that the statement adheres to our policy

Accession codes, unique identifiers and any restrictions on data availability are included in the manuscript. No clinical datasets or third party data was used.

Research involving human participants, their data, or biological material

Policy information about studies with human participants or human data. See also policy information about sex, gender (identity/presentation), and sexual orientation and race, ethnicity and racism.

Reporting on sex and gender	NA
Reporting on race, ethnicity, or other socially relevant groupings	NA
Population characteristics	NA
Recruitment	NA
Ethics oversight	NA

Note that full information on the approval of the study protocol must also be provided in the manuscript.

Field-specific reporting

Please select the one below that is the best fit for your research. If you are not sure, read the appropriate sections before making your selection.

☒ Life sciences ☐ Behavioural & social sciences ☐ Ecological, evolutionary & environmental sciences

For a reference copy of the document with all sections, see nature.com/documents/nr-reporting-summary-flat.pdf

Life sciences study design

All studies must disclose on these points even when the disclosure is negative.

Sample size	Two wild types and five mutants were used for growth phenotype. One wild type and two mutants were used for qRT-PCR. Two wild types and two mutants were used for metabolome and lipidome analysis.
Data exclusions	In metabolome and lipidome analysis, one replication was excluded.
Replication	All attempts for replication were successful.
Randomization	Allocations in this study are random.
Blinding	The blinding was not needed as the plants were grown under highly controlled conditions.

Reporting for specific materials, systems and methods

We require information from authors about some types of materials, experimental systems and methods used in many studies. Here, indicate whether each material, system or method listed is relevant to your study. If you are not sure if a list item applies to your research, read the appropriate section before selecting a response.

Materials & experimental systems

n/a	Involved in the study	
☐	☒	Antibodies
☒	☐	Eukaryotic cell lines
☒	☐	Palaeontology and archaeology
☒	☐	Animals and other organisms
☒	☐	Clinical data
☒	☐	Dual use research of concern
☐	☒	Plants

Methods

n/a	Involved in the study	
☒	☐	ChIP-seq
☒	☐	Flow cytometry
☒	☐	MRI-based neuroimaging

Antibodies

Antibodies used	Anti PGDH antibody prepared using recombinant Arabidopsis PGDH1 protein.

Validation	The PGDH antibody reacted to all of recombinant PGDH proteins, but with different affinities. This antibody is prepared by Yoshida et al (https://doi.org/10.1074/jbc.RA120.014263).

Dual use research of concern

Policy information about dual use research of concern

Hazards

Could the accidental, deliberate or reckless misuse of agents or technologies generated in the work, or the application of information presented in the manuscript, pose a threat to:

No	Yes	
☒	☐	Public health
☒	☐	National security
☒	☐	Crops and/or livestock
☒	☐	Ecosystems
☒	☐	Any other significant area

Experiments of concern

Does the work involve any of these experiments of concern:

No	Yes	
☒	☐	Demonstrate how to render a vaccine ineffective
☒	☐	Confer resistance to therapeutically useful antibiotics or antiviral agents
☒	☐	Enhance the virulence of a pathogen or render a nonpathogen virulent
☒	☐	Increase transmissibility of a pathogen
☒	☐	Alter the host range of a pathogen
☒	☐	Enable evasion of diagnostic/detection modalities
☒	☐	Enable the weaponization of a biological agent or toxin
☒	☐	Any other potentially harmful combination of experiments and agents

Plants

Seed stocks	Tak-1 and Tak-2 are wild-type Marchantia from Laboratory of Plant Molecular Biology, Kyoto University, Japan. Gemmae of the Mppgdh mutant are stocked in Metabolic System Research Team, RIKEN.
Novel plant genotypes	MpPGDH knock-out mutants were generated by CRISPR/Cas9 system with guide RNA1 ((5'-GCGCATCGTGCAAGACCGCG-3') and guide RNA2 (5'-TGAAGGCCGGTAAGTGCGCT-3'). Detail information was listed in Supplementary Figure 1 and Methods.
Authentication	The low mRNA and no protein expression level of our target gene were confirmed by qRT-PCR and Western Blot respectively in Mppgdh mutants (Supplementary Figure 1). And the mutants showed stable and obvious phenotypes compared with wild type.
